# Reduction of Preoperative Waiting Time Before Urgent Surgery for Patients on P2Y_12_ Inhibitors Using Multiple Electrode Aggregometry: A Retrospective Study

**DOI:** 10.3390/jcm9020424

**Published:** 2020-02-04

**Authors:** Michaël Hardy, Camie Dupuis, Anne-Sophie Dincq, Hugues Jacqmin, Thomas Lecompte, François Mullier, Sarah Lessire

**Affiliations:** 1 Haematology Laboratory, NAmur Research Institute for LIfe Sciences (NARILIS), Namur Thrombosis and Hemostasis Center (NTHC), Université catholique de Louvain, CHU UCL Namur, 5530 Yvoir, Belgium; 2 Department of Anesthesiology, NAmur Research Institute for LIfe Sciences (NARILIS), Namur Thrombosis and Hemostasis Center (NTHC), Université catholique de Louvain, CHU UCL Namur, 5530 Yvoir, Belgium; 3 Departments of Medicine, University Hospitals of Geneva, and GpG-Faculty of Medicine Geneva University, 14 CH-1211 Geneva, Switzerland

**Keywords:** multiple electrode aggregometry, multiplate, P2Y_12_ inhibitors, clopidogrel, prasugrel, ticagrelor, preoperative, perioperative, urgent surgery

## Abstract

P2Y_12_ inhibitor discontinuation is essential before most surgical interventions to limit bleeding complications. Based on pharmacodynamic data, fixed discontinuation durations have been recommended. However, as platelet function recovery is highly variable among patients, a more individualized approach based on platelet function testing (PFT) has been proposed. The aim of this retrospective single-centre study was to determine whether PFT using whole blood adenosine diphosphate–multiple electrode aggregometry (ADP–MEA) was associated with a safe reduction of preoperative waiting time. Preoperative ADP–MEA was performed for 29 patients on P2Y_12_ inhibitors. Among those, 17 patients underwent a coronary artery bypass graft. Twenty one were operated with an ADP–MEA ≥ 19 U (quantification of the area under the aggregation curve), and the waiting time was shorter by 1.6 days (median 1.8 days, IQR 0.5–2.9), by comparison with the current recommendations (five days for clopidogrel and ticagrelor, seven days for prasugrel). Platelet function recovery was indeed highly variable among individuals. With the 19 U threshold, high residual platelet inhibition was associated with perioperative platelet transfusion. These results suggest that preoperative PFT with ADP–MEA could help reduce waiting time before urgent surgery for patients on P2Y_12_ inhibitors.

## 1. Introduction

P2Y_12_ inhibitors are antiplatelet drugs recommended for secondary thrombosis prophylaxis after coronary, cerebral, or peripheral artery thrombosis [1]. However, residual P2Y_12_ receptor inhibition has been associated with postoperative bleeding, blood transfusion, and re-exploration for bleeding after cardiac surgery [2]. Therefore, preoperative discontinuation of these drugs is mandatory before most surgical interventions.

Based on pharmacokinetic data, current guidelines recommend five days of discontinuation for clopidogrel and ticagrelor and seven days of discontinuation for prasugrel before elective surgery to avoid significant residual antiplatelet effect [3,4]. Although clopidogrel and prasugrel are prodrugs that both irreversibly inhibit the P2Y_12_ subtype of adenosine diphosphate (ADP) receptor, the latter achieves a greater inhibition, requiring a longer discontinuation duration. Ticagrelor and one of its active metabolites act directly and reversibly, but inhibit strongly the P2Y_12_ ADP receptor, therefore also requiring a preoperative discontinuation of five days.

Nevertheless, recovery of platelet function after P2Y_12_ inhibitor discontinuation is highly variable among individuals, with some patients recovering much sooner a platelet reactivity deemed sufficient to face a haemostatic challenge [5,6,7]. As a shorter preoperative waiting time can be beneficial for the patient in case of urgent surgery, monitoring of platelet function represents a good tool to decide which patients could benefit from an earlier surgery. Therefore, the 2011 clinical practice guidelines on blood conservation of the Society of Thoracic Surgeons and the Society of Cardiovascular Anesthesiologists, and the 2018 communication from the Scientific and Standardization Committee of the International Society on Thrombosis and Haemostasis about laboratory monitoring of P2Y_12_ inhibitors suggest the use of platelet function tests (PFT) to identify the patients who could benefit from an earlier operative coronary revascularization (grade II recommendations) [8,9]. However, there is still a lack of evidence to support these recommendations [10].

Several devices analyzing platelet function are available in clinical practice. Their characteristics will not be discussed here [11]. Even if their sensitivity to evaluate the effect of antiplatelet drugs is debated, these biomarkers have been associated with postoperative bleeding and transfusion in a growing number of studies [12]. To date, only Platelet Function Analyzer 100^®^ (PFA-100) and Thromboelastography^®^ with Platelet Mapping^TM^ (TEG-PM) have been investigated in clinical studies to estimate the optimal timing to perform surgery in patients receiving clopidogrel [13,14]. In that setting, it remains to assess multiple electrode aggregometry (MEA) as a candidate PFT, and the other P2Y_12_ inhibitors (prasugrel and ticagrelor), associated with less pharmacokinetic variability [15]. MEA evaluates platelet function by detecting electrical impedance modifications when platelets aggregate and deposit on electrodes. ADP is used as an activator to evaluate response dependent on P2Y ADP receptors [16]. ADP–MEA has been associated with bleeding outcomes after cardiac surgery in patients taking P2Y_12_ inhibitors [12], and could therefore represent a good tool to identify which patients could benefit from an earlier surgery.

The aim of this study was to evaluate whether preoperative platelet function evaluation using ADP–MEA is associated with a reduction in preoperative waiting time before urgent cardiac and non-cardiac surgeries in patients receiving P2Y_12_ inhibitors.

## 2. Patients and Methods

This retrospective study was approved by the Ethics Committee of the CHU UCL Namur (NUB: B039201938718). It was conducted at the CHU UCL Namur, Godinne site.

Between January 2012 and December 2018, we included patients on P2Y_12_ inhibitors (clopidogrel, prasugrel, ticagrelor) who have had a platelet function assessment by a Multiplate^®^ analyzer (Roche diagnostics, Basel, Switzerland) using adenosine diphosphate (ADP) as activator to evaluate residual antiplatelet drug effects before surgery. Exclusion criteria were unknown date of last drug intake and first ADP–MEA performed more than seven days after P2Y_12_ inhibitor discontinuation. Only the last preoperative MEA value was included in the analysis of the association with clinical outcomes when successive analyses were performed for the same patient. In our institution, timing of urgent surgery in patients on antiplatelet therapy is determined collegially with the referent anaesthesiologist and surgeon, based on residual platelet activity and urgency of surgery, rather than on a predefined threshold proposed in the literature. We recorded the following items for each patient: sex, age, height, weight, renal function (creatinine clearance by Cockcroft–Gault formula), hepatic function (presence of cirrhosis), type of antiplatelet drug taken and last intake time, preoperative haemoglobin level, preoperative platelet count, Multiplate^®^ timing and value, type of surgery and incision time, reoperation for bleeding, blood products transfused during the hospital stay, mortality.

Whole blood was collected by venepuncture into 3 mL plastic tubes containing hirudin as anticoagulant (Roche diagnostics, Basel, Switzerland). Manual transportation to the central laboratory was used to avoid the alteration of MEA results due to the use of pneumatic tubes [17,18]. Platelet function was assessed using a Multiplate^®^ analyzer within three hours after blood collection and after a resting time of at least 15 min. Details of this method have been previously described [16]. Whole blood samples were diluted with NaCl 0.9% 1:1 v/v and activated with ADP (provided by Roche, final concentration: 6.5 μM). The modification of impedance was quantified by arbitrary aggregation units (AU), which were plotted against time to obtain an aggregation curve. Platelet function was quantified by the area under the aggregation curve (AUC (10 AU*min = 1 U), local reference range (determined with 39 healthy volunteers by a non-parametric method): 36‒93 U).

The primary outcome was defined as the difference between the recommended and observed time-intervals between last P2Y_12_ inhibitor intake and surgical incision. In this analysis, we considered patients for whom a predetermined ADP–MEA threshold of 19 U had been reached before surgery. This threshold has been determined in a large prospective trial focusing PCI patients [19], and has been proposed in a recent review from Mahla et al. [12].

Regarding secondary outcomes, we first examined the relation between time since last P2Y_12_ inhibitor intake and MEA value. Then, we evaluated frequency of severe bleeding in the whole cohort, as defined by thrombolysis in myocardial infarction (TIMI) bleeding criteria (TIMI bleeding in the setting of coronary artery bypass grafting (CABG) for cardiac surgery patients and major non-CABG related TIMI bleeding for non-cardiac surgery patients) [20]. We also explored whether the ADP–MEA value was predictive of perioperative platelets or packed red blood cells (PRBC) transfusions, and whether the 19 U threshold was predictive of perioperative platelets or PRBC transfusions.

Unless otherwise specified, data are expressed as median (interquartile range) for quantitative data and as counts for qualitative data. Difference between recommended (fixed) and observed time-intervals between the last P2Y_12_ inhibitor intake and surgery was tested different from zero using a one sample t-test. Categorical variables were compared using the chi-squared test or Fisher’s exact test, as appropriate. Based on their distributions, continuous variables were compared by independent samples t tests or Mann–Whitney U tests. All statistical tests were two-sided and a *p*-value < 0.05 was considered as significant. Statistical analysis was performed in R, version 3.4.4 [21].

## 3. Results

### 3.1. Patients’ Characteristics

**Figure 1:**
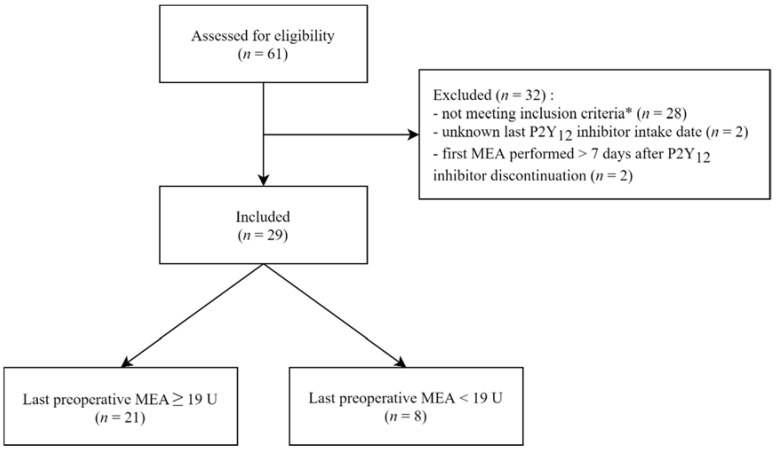
Patient flow diagram. Last preoperative multiple electrode aggregometry (MEA) test was performed 3.4 h (IQR: 0.7‒19.2) before surgery. * MEA not performed for a patient taking a P2Y_12_ inhibitor or not realized before an invasive procedure.

A total of 61 patients have been assessed for eligibility. Among these, we included 29 patients; reasons for non-inclusion are detailed in Figure 1. Twenty one of them were operated upon with a last preoperative ADP–MEA value ≥19 U, and eight of them with a last preoperative ADP–MEA value <19 U because the surgery was judged as non-deferrable despite substantial residual P2Y_12_ inhibitor effect. Median time between P2Y_12_ inhibitor discontinuation and surgery was 2.8 days (1.3‒4.0).

Demographic and perioperative data of study patients are presented in Table 1. Median time between last preoperative MEA and surgery was 3.4 h (0.7‒19.2). Median baseline platelet count was 263 G/L (min-max range: 111‒381). More than half of the patients underwent CABG with CPB. Most patients had a dual antiplatelet therapy with clopidogrel and acetylsalicylic acid (ASA). Two patients received three antiplatelet drugs within the seven preoperative days (ASA and prasugrel, replaced by ASA and clopidogrel five days before surgery for the first patient, ASA and clopidogrel, replaced by ASA and ticagrelor two days before surgery for the second). Three patients also received preoperative tirofiban infusion (GPIIb/IIIa inhibitor), which had been started after percutaneous coronary intervention (PCI) and discontinued 0, 4, and 6 h preoperatively (0, 3, and 6 h before MEA analysis, respectively); they had been operated with an ADP–MEA equal to 1, 9, and 37 U, 0, 1, and 3 days after P2Y_12_ inhibitor discontinuation, respectively. ASA was maintained or discontinued for less than five days, except for four patients (including two neurosurgical patients). Median PRBC units transfused per patient were 1 (IQR 0‒2). No patient received more than two platelet concentrates perioperatively. No patient was known to have a suspected or confirmed inherited platelet function disorder.

### 3.2. Primary Outcome

For patients operated with an ADP–MEA value ≥19 U (*n* = 21), the time-interval between the last P2Y_12_ inhibitor intake and surgery was significantly shorter by 1.6 day (95% CI 0.8‒2.4), by comparison with recommendations (*p* = 0.001).

### 3.3. Secondary Outcomes

**Figure 2:**
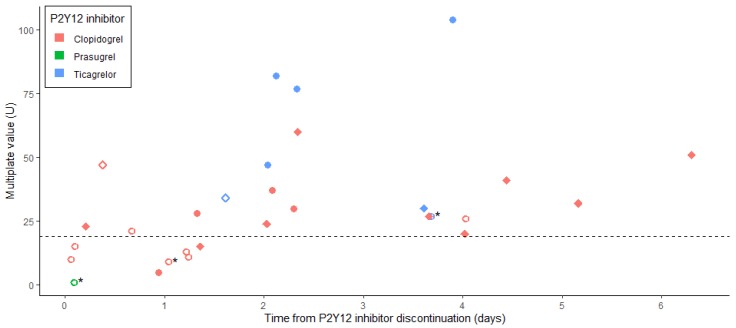
Last preoperative ADP–MEA result according to the duration of P2Y_12_ inhibitor discontinuation. Results for cardiac surgery patients are represented as circles and those for non-cardiac surgery patients as diamonds. Results for patients who have received platelet transfusion perioperatively are represented with open symbols. Dotted line represents the quantification of the area under the aggregation curve at 19 U, considered as the clinically relevant threshold. Only the last P2Y_12_ inhibitor received before surgery was represented. Asterisks represent patients who received tirofiban preoperatively.

Figure 2 represents platelet function, assessed with ADP–MEA, according to the duration of P2Y_12_ inhibitor discontinuation. We observed that platelet function recovery was highly variable for a similar duration of antiplatelet agent (APA) discontinuation. Among patients having recovered before surgery a platelet function deemed sufficient to face the haemostatic challenge (defined by an ADP–MEA value ≥19 U, *n* = 21), more than half of them did it within three days of P2Y_12_ inhibitor discontinuation (*n* = 12). 

Only two patients out of the 29 experienced perioperative severe bleeding, as defined by TIMI bleeding criteria. Of note, these two patients, for whom the preoperative ADP–MEA value was >19 U (41 U and 47 U, respectively), underwent intracranial neurosurgery (stereotactic biopsy and intracranial haemorrhage following traumatic brain injury), and suffered from postoperative intracranial haemorrhage (leading to death for the first patient).

**Figure 3:**
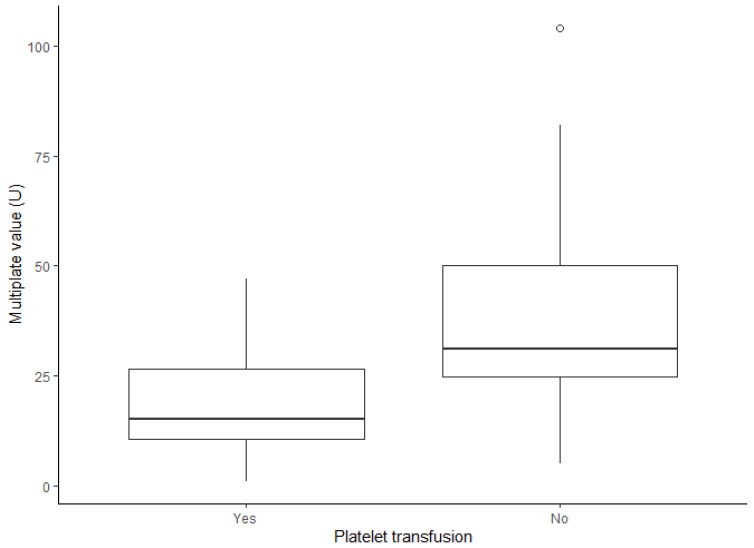
Last preoperative ADP–MEA result according to perioperative platelet transfusion status. Patients with perioperative platelet transfusion had a mean preoperative ADP–MEA value significantly lower than patients without (*p* = 0.03). Boxes represent interquartile ranges, central line represents the median, and whiskers represent the minimum–maximum range, excluding one outlier value, represented as a circle.

We identified that patients with perioperative platelet transfusion had a mean ADP–MEA value significantly lower than patients without perioperative platelet transfusion (19.4 U vs. 40.7 U, *p* = 0.01), as represented in Figure 3. Patients with perioperative PRBC transfusion had also a mean ADP–MEA value lower than patients without perioperative PRBC transfusion (29.9 U vs. 37.2 U, *p* = 0.048). The 19 U ADP–MEA threshold was predictive of perioperative platelet transfusion (RR 3.15 (1.33‒7.47), *p* = 0.03), but not of PRBC transfusion (*p* = 0.11).

## 4. Discussion

Our study suggests that assessing platelet function with ADP–MEA in patients on P2Y_12_ inhibitors before surgery is associated with a reduction of preoperative waiting time. Of note, the last preoperative ADP–MEA test was realized only a few hours before surgery in most patients (median: 3.4 h; IQR: 0.7‒19.2), which means that these values could be a good indicator of platelet function at the time of surgery. Based on a safety threshold of 19 U, a reduction of 1.6 days between the last P2Y_12_ inhibitor intake and surgery seemed safe, by comparison with the recommended discontinuation durations. The only two patients out of the 29 who experienced perioperative severe bleeding underwent intracranial neurosurgery while platelet function was found substantially corrected. On the whole, this is in line with the results of two previous studies that have found it possible to postpone urgent CABG surgery 2.3 and 1.4 days less than the recommended discontinuation durations in patients on clopidogrel using TEG^®^-PM^TM^ and PFA-100^®^, respectively [13,14]. Results are also consistent with other studies demonstrating that platelet function, assessed by MEA or VerifyNow^®^ using ADP as activator, could recover sufficiently to avoid major bleeding in most patients within three days of P2Y_12_ inhibitor discontinuation [5,7,22,23]. VerifyNow^®^ is a PFT specifically designed for the measurement of the effect of APA, which has been mainly used by cardiologists to monitor the response to these drugs. It has also been suggested as a tool to determine the optimal timing to perform CABG surgery in patients on P2Y_12_ inhibitors [12,24]. There are other candidate tests using whole blood. PFA-100^®^ (and now PFA-200^®^) has the distinct feature to operate with flowing blood; specific reagents are sensitive to P2Y_12_ inhibitors (P2Y reagents) [25,26]. Platelet mapping^TM^ is a modified TEG^®^ method to assess platelet function by measuring the strength of the clot; it has been designed to improve the poor sensitivity of TEG^®^ to platelet function disorders and antiplatelet drug effects [27].

We have also observed that recovery of platelet function is highly variable among individuals (Figure 2). This could be explained by poor observance or under-dosing in this real-life setting, but also by high basal platelet reactivity, drug interactions, increased platelet turn-over (for clopidogrel and prasugrel, which inhibit their target irreversibly) or genetic polymorphism in P2Y_12_ receptor or drug metabolism [28]. This reinforces the potential interest of PFT to identify patients who could be operated on earlier, or by contrast patients needing more time to recover a suitable platelet function (although being a situation never encountered in the present study), and those for whom platelet transfusion may be not required in first line to treat perioperative bleeding (i.e., normal platelet function, ADP–MEA > 46 or 50 U [12,29]).

An additional cause of variability is the addition of a GPIIb/IIIa inhibitor (tirofiban at our institution) to the antiplatelet regimen. The recovery of platelet activation through P2Y_12_ is difficult to assess when GPIIb/IIIa (the effector receptor for aggregation) is inhibited, even partially. Indeed, some authors stated that the effect of P2Y_12_ inhibitors cannot be adequately measured by ADP–MEA before 6.7 h after the discontinuation of tirofiban administration, and recommend waiting 12 h to perform the test [30]. As a matter of fact, a very low ADP–MEA result, whatever the reason(s), would indicate a substantial bleeding risk.

We identified an association between ADP–MEA and perioperative platelet transfusion. However this result should be interpreted with caution because of the retrospective nature of this study. This result is also limited by the fact that we have not distinguished cardiac surgery under CPB from other surgeries. Indeed, in this former setting, platelets could have been transfused because of CPB-induced platelet dysfunction causing insufficient haemostasis regardless of residual APA effect [31]. However the knowledge that platelet function had recovered to some degree could at least have spared some unnecessary platelet transfusions. This is not without interest, knowing the cost, side effects, and potential thrombotic risk associated with unnecessary platelet transfusion [32,33].

As outlined above, in our cohort, two patients suffered from severe bleeding, as defined by TIMI bleeding criteria. These patients both underwent intracranial surgery, and both experienced postoperative intracranial bleeding despite a preoperative ADP–MEA value >19 U (41 U and 47 U). This may suggest that a higher threshold could be necessary before intracranial neurosurgery. This would be consistent with other papers recommending longer anticoagulant discontinuation before neurosurgery due to the disastrous consequences of intracranial bleeding [34,35]. However, these observations have to be confirmed on a larger number of patients. Of note, ASA had been discontinued for less than five days for both patients due to the urgency of the procedure, but MEA ASPI (using arachidonic acid 0.5 mM final concentration as triggering agent) and collagen (final concentration: 6.3 µg/mL) tests were within local reference ranges, ruling out significant residual ASA effect.

The minimal platelet function on ADP–MEA needed to secure sufficient haemostatic competence and thus avoid perioperative major bleeding is not firmly established, and is likely to depend on patient’s individual factors, type of surgery, and surgeon’s experience. Typical median ADP–MEA values of patients on dual antiplatelet therapy are 22 U for clopidogrel + ASA (range 0‒91) [36], 12 U for prasugrel + ASA (IQR 8‒22), and 12 U for ticagrelor + ASA (IQR 7‒21) [37]. However, the effect of dual antiplatelet therapy might not be well integrated because ADP–MEA is little influenced by low doses of ASA [38]. The 19 U threshold used in this study and proposed by Mahla et al. [12] is the most strongly validated in clinical studies, but only in PCI settings [19]. For cardiac surgery, different cut-offs of ADP–MEA predicting bleeding risk have been identified for patients on P2Y_12_ inhibitors, ranging from 22 U to 73 U, but none has been validated in large prospective randomized control trials [39,40,41,42,43,44,45]. The variability of proposed haemorrhagic thresholds can be explained by different definitions of bleeding, different populations, small number of patients, and different pre-analytical conditions. Indeed, the time-interval between blood sampling and analysis was often not precisely specified in those clinical trials, and different anticoagulants have been used in sample tubes intended for MEA analysis (although hirudine is recommended, classical anticoagulation with citrate have been sometimes used). This uncertainty about safety thresholds led to the definition of a grey area where platelet function has recovered to some degree, but possibly not enough to safely perform elective surgery, at least the most at risk for bleeding. For example Mahla et al. [12] have defined an uncertainty area between low platelet reactivity (ADP–MEA < 19 U) and high platelet reactivity (ADP–MEA > 46 U), and suggest, if possible, to wait until platelet function has recovered up to 46 U to perform cardiac surgery. For non-cardiac surgery, no threshold has been studied to the best of our knowledge. We have thus arbitrarily chosen the same threshold as for the cardiac surgery population.

In our study, no patient received direct oral anticoagulants (DOAC). However, the association between DOACs and P2Y_12_ inhibitors is becoming more and more frequent, as recent data suggest that this attitude is associated with fewer bleeding complications, by comparison with the association between vitamin K antagonists and APAs [46,47,48,49]. Adding a DOAC to dual antiplatelet therapy increases bleeding risk [50]. This situation is not properly evaluated by MEA, which is insensitive to the effect of DOACs [51,52,53]. Further research should therefore focus on the perioperative management of patients taking both potent APAs and DOACs and scheduled for urgent invasive procedures.

This study has several limitations. First, it is a small retrospective study and subgroup or stratified analyses were not possible due to limited amount of available data. Indeed, we could not distinguish cardiac and non-cardiac surgeries, which represent different situations with specific issues. Moreover, we could not analyze separately the three inhibitors, which have distinct characteristics. Second, we only investigated the occurrence of major bleeding, as defined by TIMI criteria, in the setting of urgent surgery. We do not assume that this threshold is high enough to avoid any non-major bleeding. The decision on the optimal timing to perform surgery should therefore be multidisciplinary and take into account the urgency of surgery and the comorbidities of the patient. Finally, platelet function is only one aspect of haemostasis. Our analysis would have been stronger if we could have accounted for potential confounders such as patient’s comorbidities, concomitant medications, or type of surgery.

Given the variability of recovery of platelet function after APA discontinuation, this study adds to the existing evidence that it is possible to reduce preoperative waiting time before urgent surgery by assessing platelet function with MEA for patients on P2Y_12_ inhibitors. However, the proposed thresholds are not yet validated enough neither to guaranty sufficient haemostasis nor to precisely predict transfusion need. Moreover, some surgeries, such as neurosurgery (see the two cases in this study), are likely to require a more complete recovery of platelet function than others. Overall, the decision of timing urgent surgery in patients on P2Y_12_ inhibitors should be based on an individualized attitude, taking into account patient’s characteristics and type of surgery, and for which the objective, reliable, and relevant assessment of platelet function could be of value.

## Figures and Tables

**Table 1:** Patients characteristics (*n* = 29).

Variable	Median (IQR) or Count (Total Number)
**Sex** (male)	20 (29)
**Age** (y)	69 (62; 80)
**Height** (cm)	172 (165; 175)
**Weight** (kg)	75 (70; 90)
**Renal failure** (CrCl < 30 mL/min)	2 (29)
**Cirrhosis**	0 (29)
**APA**	
Acetylsalicylic acid	28 (29)
Clopidogrel	22 (29)
Prasugrel	2 (29)
Ticagrelor	7 (29)
Tirofiban	3 (29)
**Preoperative haemoblobin (g/dL)**	12.30 (10.30; 13.70)
**Preoperative platelet count (G/L)**	263 (195; 306)
**Last preoperative ADP–MEA value (U)**	27 (15; 41)
**Time from P2Y_12_ inh discontinuation to MEA (d)**	2.04 (1.04; 3.67)
**Time from P2Y_12_ inh discontinuation to surgery (d)**	2.82 (1.27; 3.96)
**Time from MEA to surgery (d)**	0.14 (0.03; 0.80)
**Surgery**	
CABG	17 (29)
Neurosurgery	4 (29)
Orthopaedic	3 (29)
Thoracic	2 (29)
Abdominal	1 (29)
Vascular	1 (29)
Catheter ablation of VF	1 (29)
**Reoperation for bleeding**	2 (29)
**PRBC transfusion (nb of patients)**	
Preoperative	2 (29)
Peroperative	8 (29)
Postoperative	13 (29)
**FFP transfusion (nb of patients)**	
Preoperative	0 (29)
Peroperative	3 (29)
Postoperative	2 (29)
**Platelet transfusion (nb of patients)**	
Preoperative	1 (29)
Peroperative	11 (29)
Postoperative	1 (29)

ADP: adenosine diphosphate, APA: antiplatelet agent, MEA: multiple electrode aggregometry, CABG: coronary artery bypass grafting, VF: ventricular fibrillation, PRBC: packed red blood cells, FFP: fresh frozen plasma, CrCl: creatinine clearance (Cockcroft–Gault formula).
